# *Stenotrophomonas* strain CPCC 101271, an intestinal lifespan-prolonging bacterium for *Caenorhabditis elegans* that assists in host resistance to *“Bacillus nematocida”* colonization

**DOI:** 10.1007/s00203-021-02467-4

**Published:** 2021-07-14

**Authors:** Rui Han, Yu Wang, Yang Deng, Yuqin Zhang, Lin Zhang, Qiuhong Niu

**Affiliations:** 1 College of Life Science and Agricultural Engineering, Nanyang Normal University, 1638 Wolong Road, Nanyang, 473061 Henan China; 2 Institute of Medicinal Biotechnology, Chinese Academy of Medical Sciences & Peking Union Medical College, Beijing, 100050 China

**Keywords:** *Stenotrophomonas*, *Caenorhabditis elegans*, *"Bacillus nematocida"*, Lifespan-prolonging, Colonization

## Abstract

The soil-dwelling, opportunistic pathogenic bacterium *"Bacillus nematocida"* B16 exhibits strong killing activities against a variety of pathogenic nematodes via a “Trojan horse” mechanism that can kill worm species like *Caenorhabditis elegans*. The bacterial strain CPCC 101271 was previously isolated from the intestines of *C. elegans* that were recovered from natural habitats and can serve as a probiotic for *C. elegans*, while also assisting in resistance to infection by the pathogenic strain B16. In this study, the lifespan of *C. elegans* fed with strain CPCC 101271 cells was extended by approximately 40% compared with that of worms fed with *Escherichia coli* OP50 cells. In addition, the colonization of *C. elegans* by the pathogenic bacterium *"B. nematocida"* B16 was inhibited when pre-fed with strain CPCC 101271. Metagenomic sequence analysis of intestinal microbiota of *C. elegans* fed with strain CPCC 101271 and infected with B16 revealed that pre-feeding worms with CPCC 101271 improved the diversity of the intestinal bacteria. Moreover, community structure significantly varied in coordination with *Stenotrophomonas* spp. and *Bacillus* spp. abundances when competition between strains CPCC 101271 and B16 was evaluated. In conclusion, the nematode microbiota strain CPCC 101271 assisted in its host resistance to colonization by the pathogen *"Bacillus nematocida"* and can also promote life span-prolongation in *C. elegans.* These results underscore that understanding the interactions between *C. elegans* microbiota and pathogens can provide new insights into achieving effective biological control of agricultural pests.

**Supplementary Information:**

The online version contains supplementary material available at 10.1007/s00203-021-02467-4.

## Introduction

Nematodes are one kind of the most abundant worms and have a significant global impact on ecosystems, economies, agriculture, and human health. Plant parasitic nematodes cause huge economic losses to agriculture and forestry every year. It is of great importance to develop biological methods to control plant-parasitic nematodes, so as to deal with the environmental problems posed by chemical control methods (Duncan 1991). It has been reported that the intestinal flora in most nematodes plays an important role in host growth, physiological metabolism, and immune regulation (Nour et al. 2003; Haegeman et al. 2009). The stability of biocontrol agent activity in the field is an important factor restricting their development. Since microbiota is thought to be key to the stability of biocontrol agents, understanding the functions of the intestinal flora in nematodes is of great significance for improving the activity of biocontrol agents.

The worm *Caenorhabditis elegans* is susceptible to many of the pathogens that infect plant-parasitic nematodes (Sinha et al. 2012). Like most pathogens that infect *C. elegans*, pathogenic bacteria colonize the digestive tract and ultimately kill the nematode. In contrast, most bacteria such as *Escherichia coli* and *Bacillus subtilis* are usually not toxic to *C. elegans* (Garsin et al. 2001)*.* Thus, *C. elegans* has proven to be a useful and relatively simple model for studying the interactions between microbiota and pathogens. *C. elegans* worms are reared on bacterial cells of *E. coli* under standard laboratory conditions. Starting from early adulthood, bacterial cells colonize the intestinal lumen and re-form the entire gut microbiota (Portal-Celhay et al. 2012). However, in nature, the nematode *C. elegans* is a ‘microbivore’ because of its ability to consume various types of bacteria. To some extent, the gut microbiota of nematodes may be dominated by the bacteria that they feed on; these bacteria may shape the microbiota community structure, regulate metabolism and even alter the lifespan of the host (Han et al. 2018). In *C. elegans,* beneficial bacteria were also reported to modulate host defense responses to bacterial pathogens (Kim and Mylonakis 2012; Montalvo-Katz et al. 2013; Iatsenko et al. 2014; Dirksen et al. 2016; Berg et al. 2019; Kissoyan et al. 2019; Zimmermann et al. 2019).

In our previous study, we found that the bacterial pathogen strain *"B. nematocida"* B16 killed *C. elegans* nematodes by employing a “Trojan horse” mechanism (Niu et al. 2010). We have isolated several bacteria inside worms from various origins including soil and rotten fruit. Some bacteria, like *Phytobacter* sp. SCO41, showed inhibitory effects on pathogenic bacterium B16 (Wang et al. 2019). To explore the relationships between microbiota and pathogens of nematodes in-depth, we combined metagenomic sequencing analysis and culture-dependent methods to collect evidence. As a result of this analysis, we found that strain CPCC 101271, originally isolated from the intestinal lumen of *C. elegans* in nature, acts as a component of beneficial microbiota for *C. elegans* by extending the lifespan of the host, as well inhibiting the colonization of the host by *"B. nematocida"* B16, an opportunistic pathogen, which was previously proposed as a candidate biological control agent for nematodes (Huang et al. 2005). The description of the classification and identification of this strain on the basis of the polyphasic taxonomic data will be elaborated in the following article.

Here, we report an intestinal bacterium *Stenotrophomonas* strain CPCC 101271*,* and the results of in vitro and in vivo experiments showed that *"B. nematocida"* B16 can inhibit the growth of CPCC 101271, while strain CPCC 101271 has the ability to inhibit the colonization of *C. elegans* by B16. We also describe the variation in the microbiota community structure of *C. elegans* during competition between strain CPCC 101271 and B16.

## Materials and methods

### Acquisition of worms and bacterial strains

The location for screening nematodes is Baotianman Natural Reserve (33° 27′ 47′′ N; 111° 48′ 32′′ E), Nanyang, China. Four soil samples were collected and approximately 1000 wild-living nematodes were isolated using the Baerman funnel technique (Gray 1984). Single worms were isolated and collected under a dissecting microscope. After washing three times with aseptic M9 buffer, single nematodes were frozen, ground and their crude DNA was extracted. The nematode species was identified by diagnostic PCR using the primer pair nlp30 diagnostic for *C. elegans* (Petersen et al. 2014). The cultivation, synchronization, collection, and surface sterilization of *C. elegans* worms were performed as previously described (Niu et al. 2012, 2015, 2016).

Strain CPCC 101271 was isolated from the surface-sterilized *C. elegans* worms*,* using Luria–Bertani (LB) agar plates. The nematodes were surface-sterilized by soaking in a solution of 1% mercuric chloride and 2% antibiotic mixture (streptomycin sulfate and gentamicin) for 1 h, and then cultured on nutrient and oligotrophic agar plates to confirm successful surface sterilization (0 cfu). The surface-sterilized worms were ground, then approximately 0.1 g of homogenate was suspended in 10 mL sterilized saline solution (containing 0.85% NaCl, w/v) and mixed thoroughly. Next, about 0.2 mL of suspension was spread onto an LB agar plate. After incubation at 30 °C for 2 weeks, about 40 bacterial colonies were grown on the plate. According to the colony color and size, the colonies were randomly selected for separation and purification. Among which, a distinct pale yellowish colony was picked and transferred onto a newly prepared LB agar plate for further purification. The purified isolate of CPCC 101271 was maintained as a glycerol suspension (20%, v/v) at − 80 °C for long-term storage.

The reference strains *Stenotrophomonas rhizophila* JCM 13333^T^ and *S. bentonitica* DSM 103927^T^ were obtained from the Japan Collection of Microorganisms (https://jcm.brc.riken.jp/en/) and German Collection of Microorganisms and Cell Cultures (https://www.dsmz.de/collection/catalogue), respectively. *E. coli* strain OP50 was obtained from the Laboratory for Conservation and Utilization of Bio-resources, Yunnan University. The opportunistic pathogen strain *"B. nematocida"* B16 (= GCMCC 1128) (Huang et al. 2005) was obtained from the China General Microbiological Culture Collection Center (http://www.cgmcc.net). GFP-expressing strain B16g was constructed in our previous study (Niu et al. 2012).

### C. elegans lifespan assay

Worms *C. elegans* were maintained on NGM (Nematode Growth Medium) plates at 25 °C. The strains CPCC 101271, JCM 13333^T^ and OP50 used for measuring the worms’ lifespan were recovered from the 20% glycerol stock and were streaked onto LB agar plates and then incubated at 32 °C. A single colony was picked and incubated in 5 ml of LB at 32 °C overnight. One milliliter of the overnight culture was added to 100 ml of LB medium and shaken at 32 °C until an OD_600_ of 0.8 was reached. 200 µl of the tested bacterial culture was seeded on NGM plate, and then synchronized L4 larvae were transferred to the corresponding bacterial seeded NGM plate (Park et al. 2017). The lifespan experiment was monitored by scoring the dead worms every 10 h from 50 h until 160 h. Worms that did not respond to prodding with a platinum wire were considered dead. Those desiccated by crawling onto the edge of the housing plate were excluded from the analysis. The experiments were performed with five replicates at three different time intervals.

### In vitro bacteriostatic activity test

Each bacterial strain was separately inoculated into 5 mL of LB medium and cultured in a shaker at 32 °C, 180 r/min for 12 h. Then the culture broth was adjusted to an optical density value at 600 nm (OD_600_) of 1. Approximately 0.3 mL of CPCC 101271 culture broth was evenly spread onto an LB agar plate. Sterilized filter paper with a diameter of 5 mm was immersed in the bacterial culture for 5 min and placed onto the agar plate containing strain CPCC 101271. The plate was then incubated at 32 °C for 48 h, and the size of the inhibition zone for each sample was recorded. *E. coli* culture broth and LB medium were used as negative controls, while polymyxin B (300 IU) and rifampin (5 μg) were used as positive controls. The experiments were performed with three parallels and repeated thrice.

### Colonization capability assay

Colonization capability was assayed using approximately fifty 1-day-old adult hermaphrodite worms were placed on each plate at 25 °C following the procedures described by Aballay et al. (2000) and Niu et al. (2012) with modifications. In ‘Feeding Transfer’ experiments, the worms were transferred by hair and repeatedly washed using sterilized NaCl solution (0.85%, w/v). Three nematode treatment groups were set up. In the first group of nematodes pre-fed with CPCC 101271 then infected by B16g, the worms were transferred onto LB plates containing a low concentration (10^6^ cells/mL) of CPCC 101271 and co-cultivated for 4 h. The worms were then removed from the plates, washed twice, transferred to plates containing B16g and co-cultivated for 72 h. In the second group of nematodes pre-fed with JCM13333^T^ then infected by B16g, the worms were first seeded on an LB agar plate containing JCM13333^T^ (10^6^ cells/mL) and cultivated for 4 h, and then the worms were transferred to plates containing B16g and cultivated for 72 h. In the third group, the worms were first fed on the same concentration of OP50 for 4 h, then transferred to B16g plates and cultivated for 72 h. The control group of nematodes without being pre-fed with bacteria were directly seeded on a blank medium and then cultivated for 4 h before being transferred to B16g plates, which were also defined as B16 direct infection group. The colonization process was observed under a Nikon 800 Eclipse microscope (Nikon Corp., Japan) equipped for epifluorescence with a mercury lamp and an excitation filter of 450–490 nm (blue light) and a barrier filter of 515 nm. At each time point, three sets of 10 nematodes were randomly selected to evaluate colonization. The worms with fluorescent bacteria in the entire lumen were scored as full; worms without any green fluorescence signal in the lumen were scored as undetected, and worms between these two extremes were scored as partial. The worms were considered dead when no movement was observed under a light-dissecting microscope, and when gently tapping of nematodes by a platinum wire, no movement occurred. Dead nematodes whose bodies were decomposed were excluded from the analysis. The number of worms killed in each group was counted every 12 h within 72 h from B16 infection. Mortality rates of B16-infected nematodes were defined as the ratio of dead nematodes to tested nematodes. The experiments were performed thrice.

### DNA preparation and metagenomic analysis of microbiota

The tested nematodes were divided into five groups as follows: (I) CW00h group, which was pre-fed with CPCC 101271 for 4 h; (II-V) CW04h, CW08h, CW12h and CW16h groups, which were separately co-cultivated with B16 for 4, 8, 12 h and 16 h, respectively, after being pre-fed with CPCC 101271 for 4 h.

The worms were collected and then washed and surface sterilized as described above. Total DNA was extracted from the intestinal microbes using the PowerSoil DNA Isolation Kit (MoBio, USA) according to the manufacturer’s protocols. The concentration and purity of extracted DNA were determined using a TBS-380 and NanoDrop2000, respectively. The quality of the extracted DNA was evaluated on a 1% agarose gel. DNA was fragmented to an average size of about 300 bp using a Covaris M220 (Gene Company Limited, China) for paired-end library construction. The paired-end library was constructed using NEXTFLEX^®^ Rapid DNA-Seq (Bioo Scientific, Austin, TX, USA). Adapters containing the full complement of sequencing primer hybridization sites were ligated to the blunt ends of the fragments. Paired-end sequencing was performed on an Illumina NovaSeq (Illumina Inc., San Diego, CA, USA) at Majorbio Bio-Pharm Technology Co., Ltd. (Shanghai, China) using NovaSeq Reagent Kits according to the manufacturer’s instructions (www.illumina.com). Adapter sequences were stripped from the 3' and 5' ends of paired-end Illumina reads using SeqPrep (https://github.com/jstjohn/SeqPrep). Low-quality reads (length < 50 bp, a quality value < 20, or containing N bases) were removed using Sickle (https://github.com/najoshi/sickle).

Metagenomics data were assembled using MEGAHIT (https://github.com/voutcn/megahit) (Li et al. 2015), which makes use of succinct de Bruijn graphs. Contigs with a length ≥ 300 bp were selected as final assemblies and were used for further gene prediction and annotation (Noguchi et al. 2006; Li et al. 2008).

## Results

### Increase in *Caenorhabditis elegans* survival rate and lifespan by feeding on CPCC 101271

**Fig. 1:**
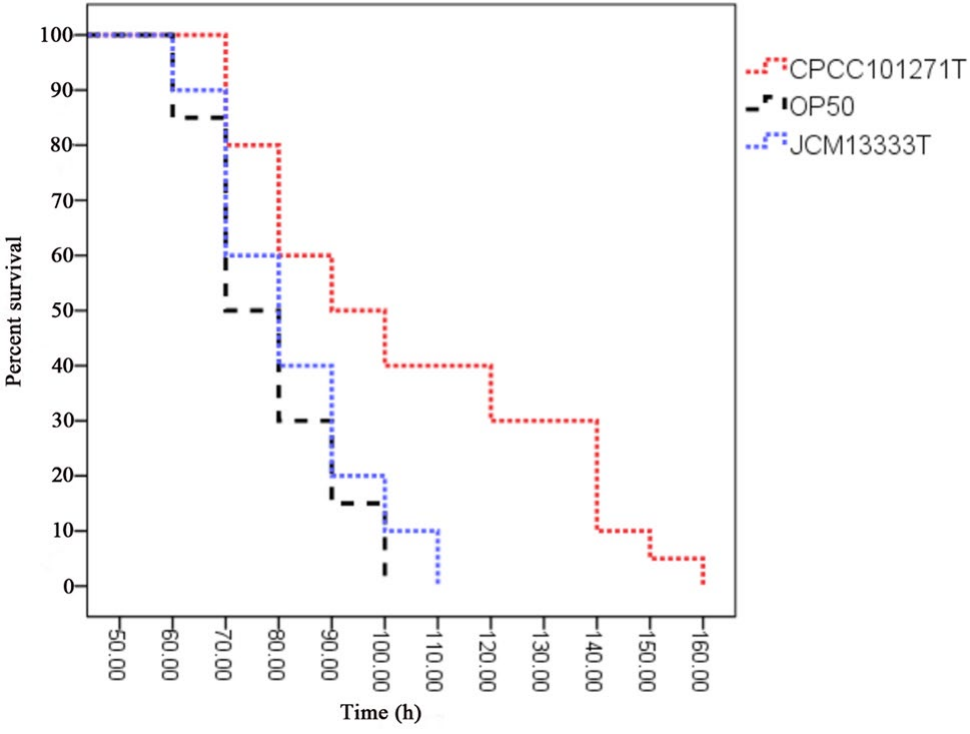
Survival rates of *Caenorhabditis elegans* fed on different bacteria

Strain CPCC 101271 was recovered from the intestinal lumen of *C. elegans* using LB agar plates. We compared the longevity of worms fed either on CPCC 101271, JCM 13333^T^ or OP50. The results showed that worms fed on JCM 13333^T^ or OP50 had almost similar lifespans. However, worms fed on CPCC 101271 lived approximately 40% longer than worms fed on *E. coli* or JCM 13333^T^, indicating that the nematodes fed on CPCC 101271 lived longer than those fed on *E. coli* OP50 or *S. rhizophila* JCM 13333^T^ and had greatly increased survival rates (Fig. 1).

### Strain CPCC 101271 confers the host with resistance to *"B. nematocida"* colonization

**Fig. 2:**
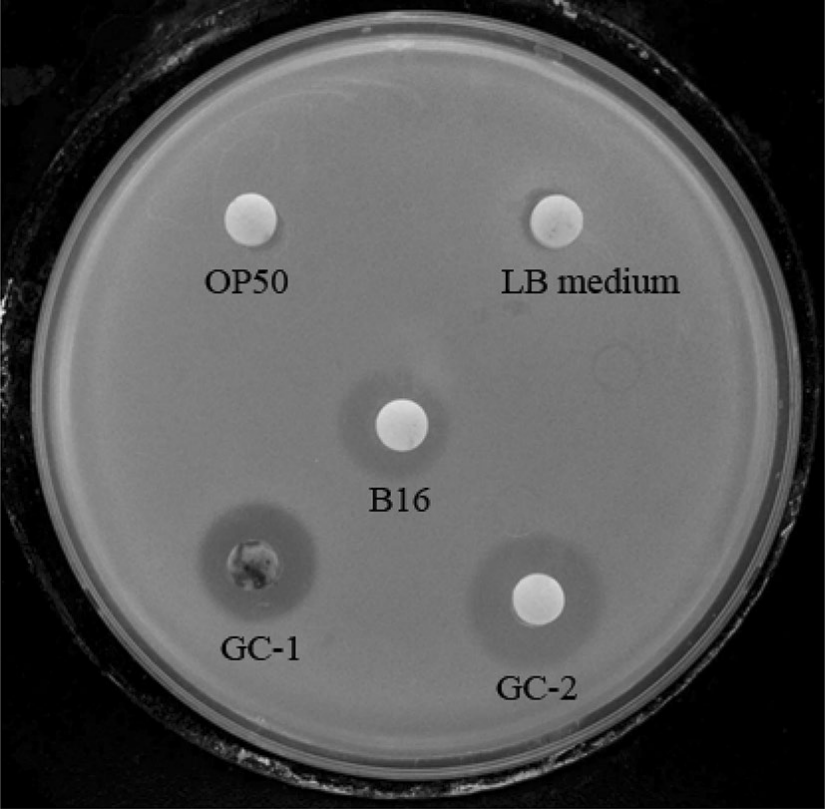
Inhibitory activity of B16 against CPCC 101271 on an LB agar plate. OP50, *E. coli* OP50; B16, *"B. nematocida"* B16; GC-1, polymyxin B (300 IU); GC-2, rifampin (5 μg)

To investigate whether strain CPCC 101271 has colonization-resistance activity against *"B. nematocida"* B16, we first performed an in vitro bacteriostatic activity test. The results showed that strain CPCC 101271 could not inhibit *"B. nematocida"* B16 but could be inhibited by B16 (Fig. 2). A transparent inhibition zone with clear edges formed around the paper containing B16 after 48 h of incubation on an LB agar plate spread with strain CPCC 101271. The clear zone, which was about 1.6 cm in diameter, was slightly smaller than the zones surrounding the positive drug controls polymyxin B (300 IU) and rifampin (5 μg). And no clear inhibition zone formed around the papers containing *E. coli* or LB medium (Fig. 2).

**Fig. 3:**
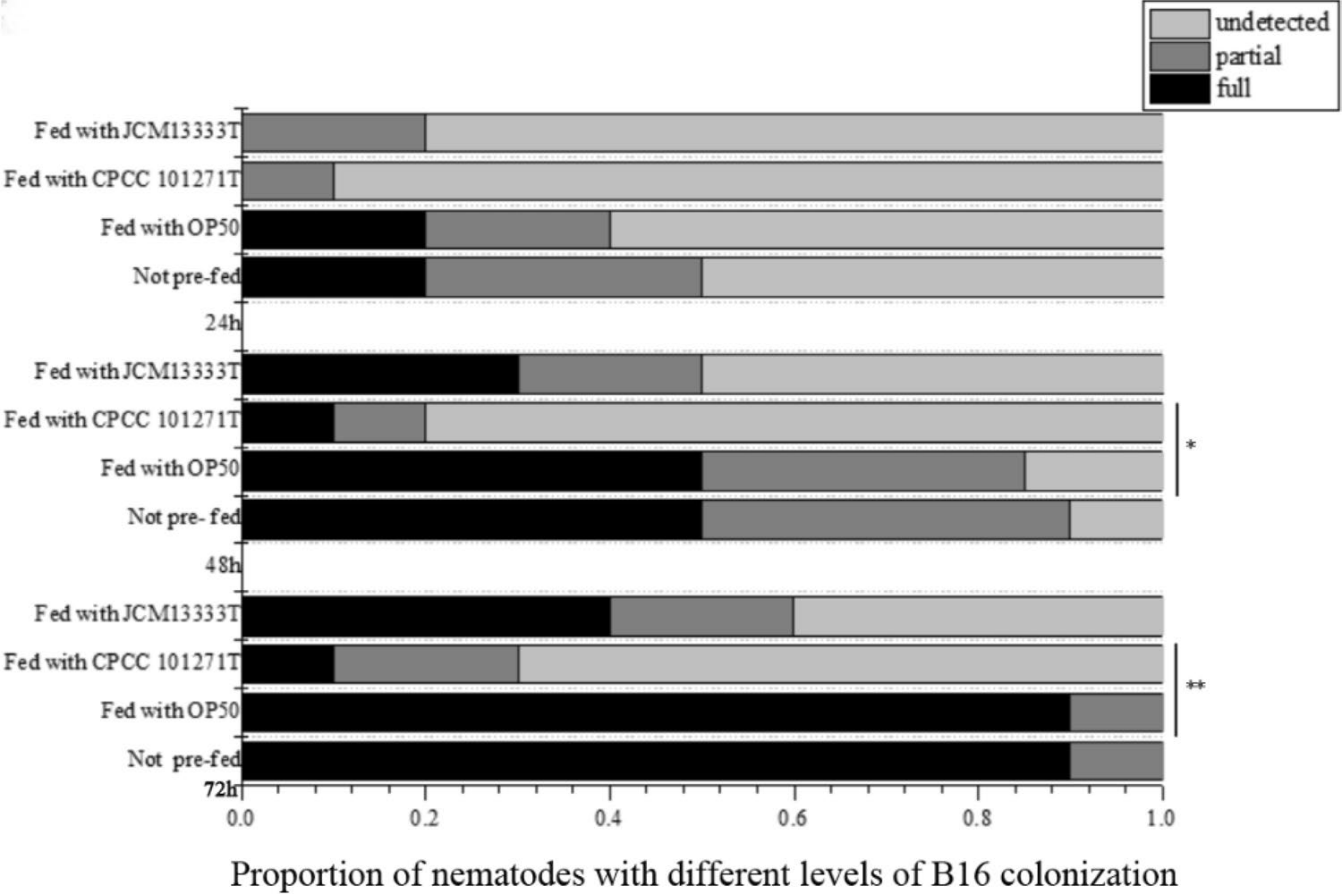
Differences in *"B. nematocida"* B16 colonization of *C. elegans* after 24, 48, and 72 h of infection. For each bacterial strain tested, the extent of colonization was scored in four sets of 10 nematodes every 24 h. A representative of three independent experiments with the average fraction of the population colonized for each category is shown. Chi-squared test, *P < 0.05, **P < 0.001. CPCC 101271, *Stenotrophomonas* strain CPCC 101271; JCM 13333^T^, *Stenotrophomonas rhizophila* JCM 13333^T^; OP50, *E. coli* OP50

GFP-expressing strain B16g was used to confirm the specificity of B16 colonization activity assays. The results of colonization-resistance activities indicated that strain CPCC 101271 could also inhibit the colonization of B16 in the nematode intestine. During the first 24 h of infection with *"B. nematocida"* B16, almost no nematodes pre-fed with CPCC 101271 were scored as being in the “full” colonization category (see Materials and Methods). By contrast, almost 20% of animals directly fed with *"B. nematocida"* B16 were scored as “full”. In addition, after infection for 48 h, 50% of the animals fed only *"B. nematocida"* B16 were scored as “full”. However, only 10% of worms pre-fed with CPCC 101271 were scored as “full” at the same time point. After 72 h, *"B. nematocida"* B16 showed notably strong colonization ability, with 90% of the worms not pre-fed with CPCC 101271 scored as “full”. In contrast, only 10% of the animals pre-fed with CPCC 101271 were scored as “full”. Moreover, compared with worms fed only B16, the percentage of worms fed with both B16 and CPCC 101271 that had undetectable B16 colonization was much higher at 48 h (10% vs. 80%) and at 72 h (0% vs. 70%)*.* The worms pre-fed with *E. coli* showed little difference compared with those in the no pre-feeding group. The ability of B16 to colonize the nematodes pre-fed with JCM 13333^ T^ was stronger than its ability to colonize those pre-fed with CPCC 101271, but a little weaker than its ability to colonize the negative controls pre-fed with *E. coli* OP50. Differences between the abilities of B16 to colonize the nematodes pre-fed with CPCC 101271 and pre-fed with OP50 were notable when we compared the changes in the severity of colonization at 72-h (Fig. 3, chi-squared test, *P* < 0.0001). For example, only 10% of worms that were pre-fed with CPCC 101271 could be categorized as having ‘full’ colonization. However, 90% of worms that were pre-fed with OP50 were categorized in the ‘full’ colonization category. The results indicated that colonization of *"B. nematocida"* B16 was markedly attenuated in *C. elegans* pre-fed with CPCC 101271.

**Fig. 4:**
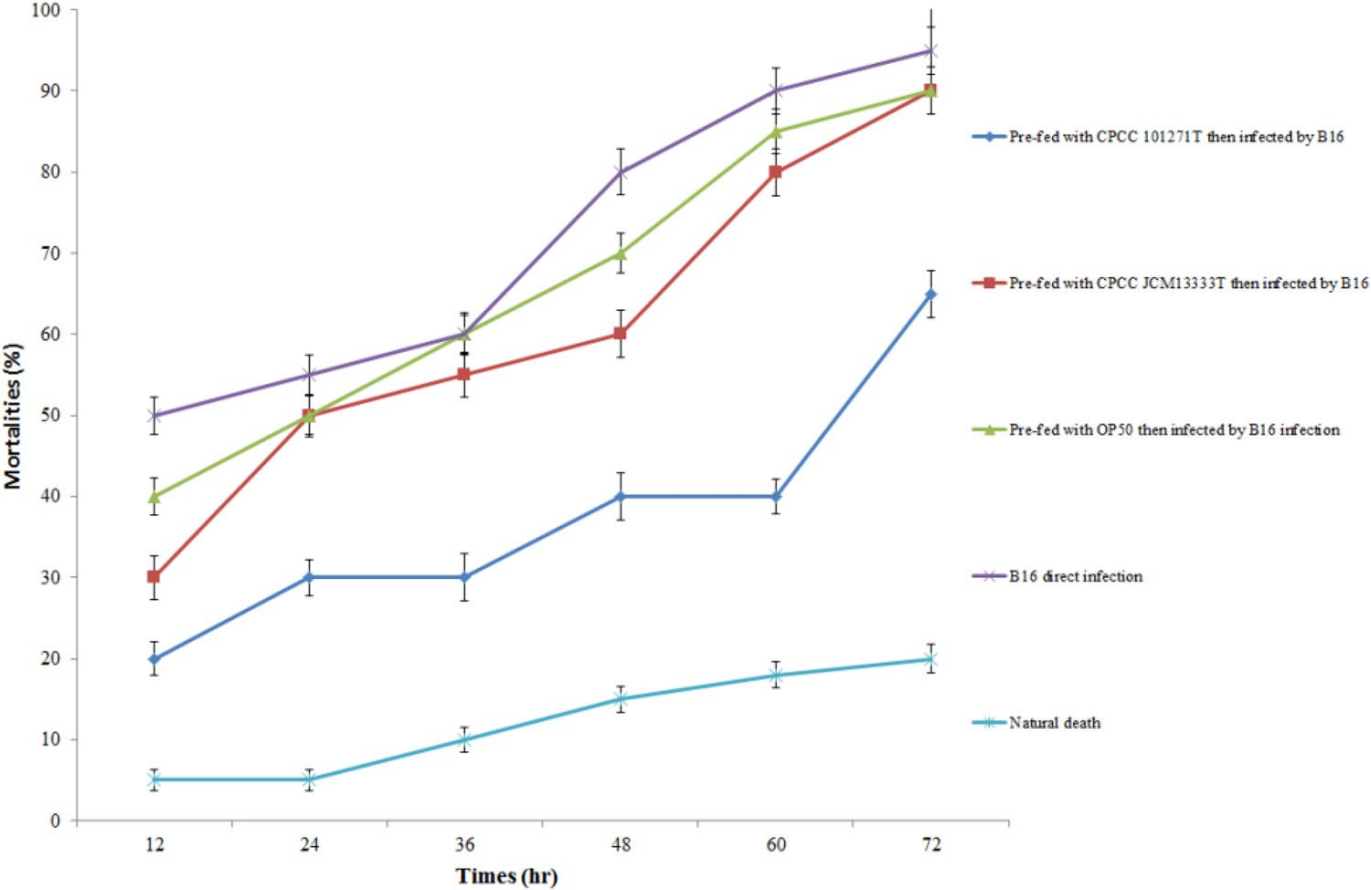
The differences in mortality rates of B16-infected nematodes in different treatment groups. CPCC 101271, *Stenotrophomonas* strain CPCC 101271; JCM 13333^T^, *Stenotrophomonas rhizophila* JCM 13333^T^; B16, *"Bacillus nematocida"* B16; OP50, *Escherichia coli* OP50

The differences in the mortalities of the B16-infected nematodes in the different treatment groups indicated that pre-feeding with CPCC 101271 reduced the mortality caused by infection with the pathogenic bacteria B16 (Fig. 4). The mortalities of the nematodes pre-fed with OP50 and then infected by B16 and the nematodes directly infected by B16 (without pre-feeding with any other bacteria) were 85 and 90% within 60 h, respectively. By contrast, for worms pre-fed with CPCC 101271 then infected with B16, the mortality dropped to 40%. The natural mortality rate of the negative control nematodes (no pre-feeding or B16 infection) was only 18%. At other time points, the mortalities of nematodes pre-fed with CPCC 101271 were significantly lower than those of nematodes pre-fed with *E*. *coli* or directly infected with B16.

### The variation in *Caenorhabditis elegans* microbiota community structure during competition between CPCC 101271 and B16

In a previous study, we collected free-living terrestrial *C. elegans* from soil and rotten fruits, and analyzed the variation in intestinal flora following *"B. nematocida"* B16 infection by performing macrogenomic analysis. We found significant differences in the diversity and distribution of microbiota between the control worms and those infected with B16 for 24 h. The diversity of the intestinal microbiome decreased after B16 infection (Niu et al. 2016). Here we aimed to investigate the variation in the intestinal bacterial community structure of *C. elegans* during competition between CPCC 101271 and B16.

**Fig. 5:**
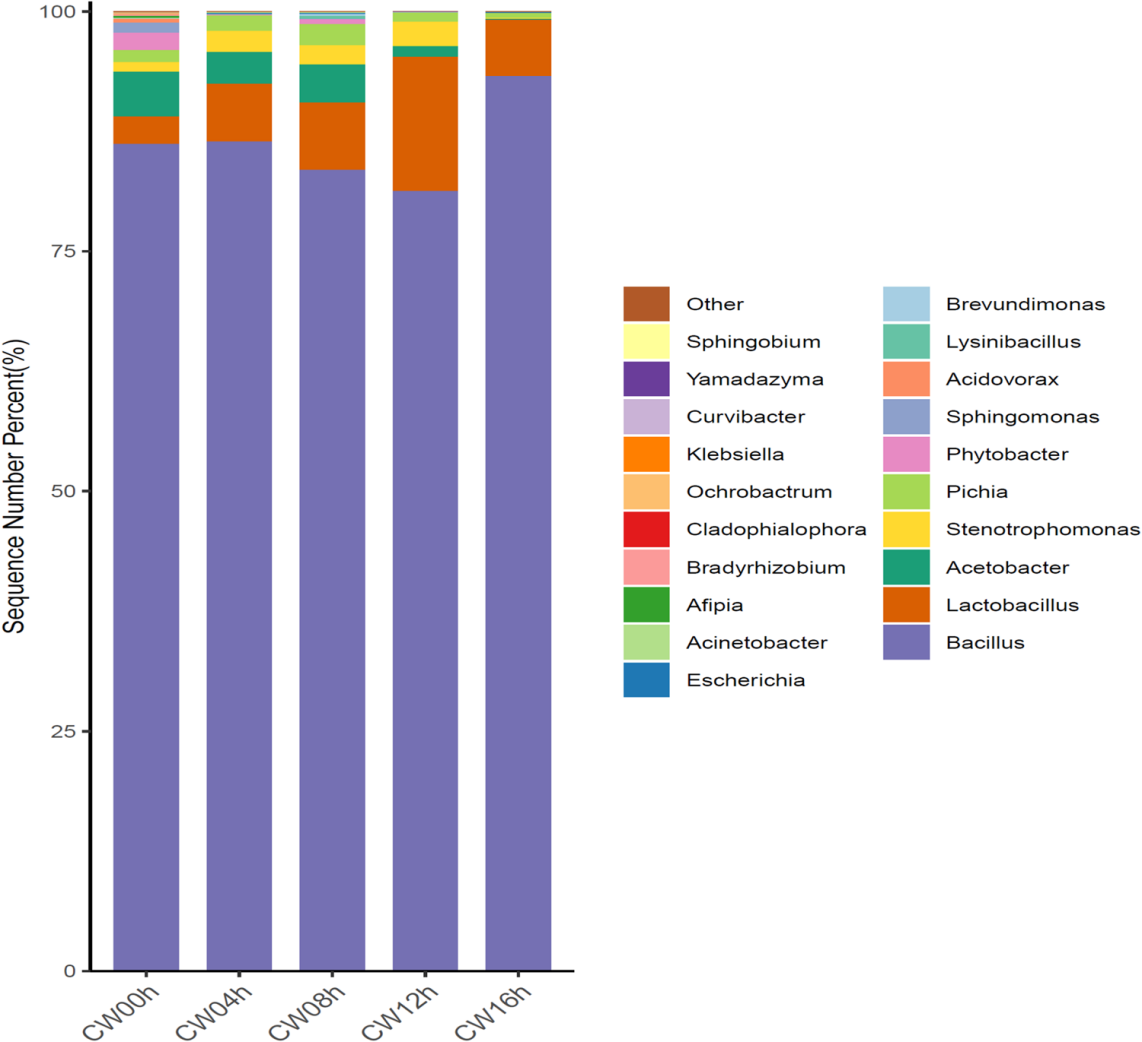
Column diagram showing the microbiota structure at the genus level, based on metagenomic sequence analysis, in nematodes pre-fed with CPCC 101271 before and after being infected by B16. CW00h, pre-fed with CPCC 101271 for 4 h; CW04h, CW08h, CW12h and CW16h, groups co-cultured with B16 for 4, 8, 12, and 16 h, respectively, after being pre-fed with CPCC 101271 for 4 h

**Fig. 6:**
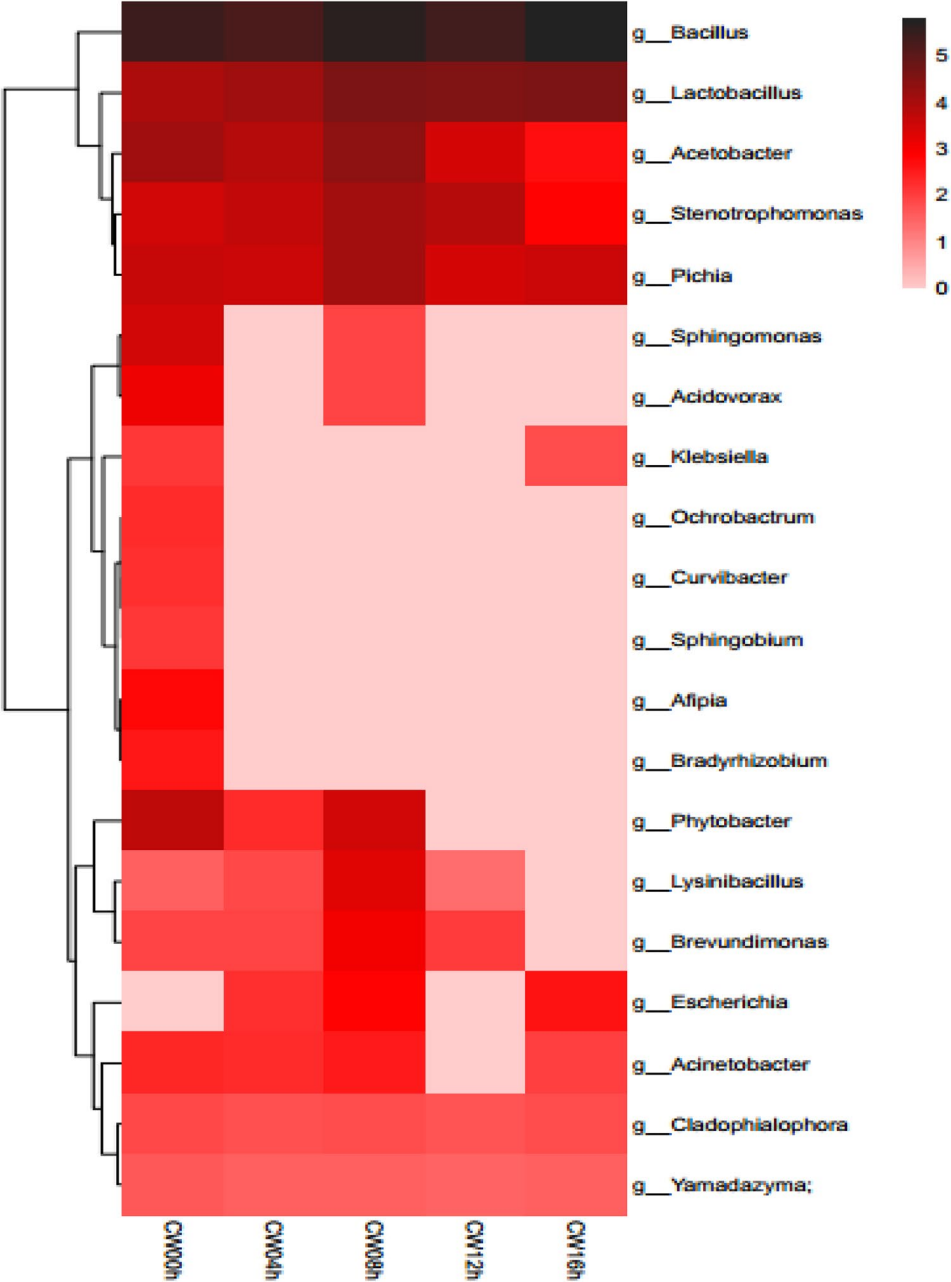
Heatmap based on metagenomic sequence analysis showing the microbiota structure at the genus level in nematodes pre-fed with CPCC 101271 before and after being infected by B16

A total of 332,314, 280,966, 705,161, 227,126 and 597,664 sequences comprising 44, 26, 49, 19 and 30 operational taxonomic units (OTUs) were obtained from the five groups CW00h, CW04h, CW08h, CW12h, and CW16h, respectively. At the genus level, these OTUs represented 26, 14, 23, 10, and 14 genera, respectively. It was obvious that the bacteria diversity was greatly decreased during competition between CPCC 101271 and B16 (Fig. 5). At the first stage of infection (CW00h) in nematodes pre-fed with strain CPCC 101271, the microbiota community structure predominantly consisted of the genera *Bacillus*, *Acetobacter*, *Lactobacillus*, *Phytobacter, Stenotrophomonas, Pichia* and *Sphingomonas*. At the second stage (CW04h), 4 h after the worms were infected by B16, dysbiosis occurred, and in the course of re-construction of the microbiota community, the bacteria diversity was drastically reduced. Besides *Lactobacillus* spp*., Acetobacter* spp*.* and *Pichia* spp., which remained the major groups, the abundance of *Bacillus* spp. increased slightly and the abundance of *Stenotrophomonas* spp. increased. At the third stage (CW08h), which we termed “the breaking period”, a large number of CPCC 101271 and B16 bacteria were co-existing and competing; the diversity of the intestinal flora had partially recovered, but the abundance of *Bacillus* spp. had greatly decreased. At the fourth stage (CW12h), the abundance of *Bacillus* spp. was even lower and the abundance of *Stenotrophomonas* spp*.* was higher. The newly reconstructed intestinal flora was disrupted again, and the species composition was the most similar to that observed at the second stage (Fig. 6). By the fifth stage (CW16h), B16 overwhelmed CPCC 101271 and only *Lactobacillus* spp. and *Pichia* spp., together with *Bacillus* spp., remained the major microbiota.

In a summary, over the course of B16 infection, the abundance of CPCC 101271 and its relatives (*Stenotrophomonas* spp*.*) kept on increasing until 8 h after B16 infection and then decreased sharply. The change in abundance of *Stenotrophomonas* spp. was accompanied by similar changes in the abundance of alpha-trehalose-phosphate synthase-encoding genes calculated from the metagenome data, except at the final stage (Figure S1). By contrast, the abundance of *Bacillus* spp*.* first decreased, then increased rapidly, which was similar to the changes in the abundance of trehalose-6-phosphate hydrolase-encoding genes (Figure S2). The abundances of alpha-trehalose-phosphate synthase-encoding genes (present in the genome of B16) and trehalose-6-phosphate hydrolase-encoding genes (present in the genome of CPCC 101271 ) together with *Stenotrophomonas* spp*.* and *Bacillus* spp. reached the highest level at 8 h (CW08h) after B16 infection. The results suggested that strain CPCC 101271 possibly participated directly or induced some other bacteria in the community to participate in resistance to B16 colonization. However, by 16 h after infection, B16 dominated the microbiota community, and the growth of CPCC 101271 was completely suppressed.

## Discussion

Gut microbiota, diverse microorganisms inhabiting the digestive track, are tightly linked to the health of their host. The community of microbial species, among which bacteria are predominant and have been extensively studied, not only generates metabolites essential for various host functions but also confers resistance to exogenous pathogens (Lee and Hase 2014). However, the molecular mechanisms by which microbiota resist pathogens and the changes in bacterial composition that occur after pathogen infection remains elusive.

It has been reported that bacteria living in most nematodes play an important role in the growth and development, physiological metabolism, and immune regulation of the host. The bacteria *Xenorhabdus* spp. and *Photorhabdus* spp. were reported to be symbionts found in the guts of nematodes including *Steinernema*, *Heteronrhahditis*, *Heterodera* and *Rhabditis.* They produce antibiotics, intracellular protein crystals, and numerous other products that help nematodes kill insects and also provide nutrients (Forst et al. 1997; Park et al. 2011; Whittaker et al. 2016; Shan et al. 2019). Bacteria associated with cysts of the soybean cyst nematode play an important ecological role in the long-term survival of cysts in soil (Nour et al. 2003). An endo-symbiotic bacterium in a plant-parasitic nematode was found to be closely associated with the growth and metabolism of its host (Haegeman et al. 2009). Therefore, to some extent, nematodes are typical symbioses with their microbiota.

For nematodes *C. elegans*, most of the research work was carried out based on the N2 strain, which has been adapted to laboratory conditions over decades (Sterken et al. 2015), including the regular and routine removal of any microbes through hypochlorite treatment. Thus N2 strain does not carry any microbes in its gut and microbiome associations are little known to the nematode *C. elegans* N2 under laboratory conditions. In contrast, worms in nature are exposed to complex microbial communities. Understanding the worm’s natural microbiome is essential to help explain their realistic and unbiased characteristics. In fact, more and more researchers have paid their attention to the natural *C. elegans* microbiome (Dirksen et al. 2016; Samuel et al. 2016; Zhang et al. 2017). A possible fitness benefit was already indicated upon gut colonization with certain non-pathogenic bacteria, leading to increased resistance against pathogens (Ikeda et al. 2007; Kim and Mylonakis 2012; Montalvo-Katz et al. 2013). However, it is yet unclear whether the beneficial bacterial isolates in *C. elegans* affect the infection effect of pathogenic bacteria on nematodes. The activities and stability of biological control agents might be effectively improved by using co-cultures of various antagonistic bacteria with different mechanisms of action and ecological adaptability. Therefore, using natural *C. elegans* as a model, studying the interactions between microbiota and biocontrol microbes is a promising approach for improving the stability of biocontrol in the field.

To date, there are 16 validly described species in the genus *Stenotrophomonas*, which have high genotypic and phenotypic diversity and were recovered from various environmental and even clinical samples (Brooke 2012). The type species *S. maltophilia* was originally recognized as a human opportunistic pathogen. Subsequent research revealed that the metabolic diversity of *S. maltophilia* is responsible for the production of novel bioactive compounds, including biocontrol agents against microbes and insects, and enzymes and nanoparticles used in medicinal, industrial, and bioremediation applications (Ribitsch et al. 2012). Another well-studied species, *S. rhizophila*, which shows an endophytic lifestyle, possesses unique genes encoding plant cell-wall-degrading enzymes and proteins responsible for the synthesis and transport of the plant-protective spermidine and high salinity tolerance, which suggests it is a harmless alternative *Stenotrophomonas* species for use in biotechnology (Alavi et al. 2013). There were also several reports on the genus *Stenotrophomonas* strains isolated from animal intestines. *Stenotrophomonas* members were found to be gut bacteria through the life cycle of the Bark Beetle *Dendroctonus rhizophagus*, and *S. maltophilia* could be implicated in nitrogen fixation and cellulose breakdown, important roles associated to insect development and fitness, especially under the particularly harsh life conditions of this beetle (Morales-Jiménez et al. 2012). Additionally, Sun et al. isolated a chitin-degradation *Stenotrophomonas* strain from the hindgut of a fungus-growing termite *Macrotermes* *barneyi* (Sun et al. 2017). The novel species studied here, a close relative of *S. rhizophila*, was generally consistent with those previously reported *Stenotrophomonas* species isolated from the nematodes sampled directly from the native habitats (Dirksen et al. 2016). In each parallel of the isolation experiment, we selected randomly 20 single natural worms isolated from the same location. Members of the genus *Stenotrophomonas* could be isolated from more than 15 worms. The *Stenotrophomonas* spp. were identified to be the same species with CPCC 101271. Furthermore, other worms without *Stenotrophomonas* being detected were raised on 9-cm agar plates seeded with 400 µl of the tested bacterium CPCC 101271 with an OD_600_ of 10 for 24 h at room temperature. Then *Stenotrophomonas* spp. could be isolated from the intestines of the worms after washed three times and surface disinfection. These experiments indicated that CPCC 101271 could stably colonize the nematode gut under experimental conditions.

In nature, the structure and diversity of the microbiota in healthy nematodes are constantly changing. Some intestinal bacteria are actually indispensable parts of the host, which may form a mutually beneficial symbiotic relationship with the host.

In this study, the interaction between the bacterium CPCC 101271 and *"B. nematocida"* B16 was investigated. When strains B16 and CPCC 101271 were co-cultured on LB plates, strain B16 showed inhibitory activities against CPCC 101271, which is consistent with the last stage of the competition between strain CPCC 101271 and B16 in worm intestine, even strain CPCC 101271 exhibited the colonization-resistance activities against *"B. nematocida"* B16 in the early stages. Firstly, strain CPCC 101271 was confirmed as probiotic to worms owning to its ability to prolong the lifespan of *C. elegans*. Secondly, strain CPCC 101271 could delay the infection time of B16 against nematodes, but not completely inhibit the infection of B16. Last but not least, *Bacillus* strains occupied the niche of *Stenotrophomonas* members by inhibiting the growth of CPCC 101271, which could be inferred from the metagenomic analysis results. And then strain B16 completed the infection and realized its proliferation in worms.

Based on the above experiments, we proposed that the reason for colonization resistance to the pathogen B16 by the strain CPCC 101271 might own to its beneficial aspects to *C. elegans*. Strain CPCC 101271 might play a critical role in (i) shaping and maintaining the intestinal bacterial community structure, (ii) synthesizing osmoprotectants, such as glucosyl glycerol and trehalose, to help maintain host homeostasis, and (iii) producing or stimulating other microorganisms to synthesize antimicrobial peptides and other stress-protective agents to protect the host from pathogens and harsh environments.

We should explore substantial evidence to confirm the above inference in the following studies. Understanding this inference mechanism can help quickly inhibit the growth of probiotic microbiota, accelerate the colonization of biocontrol bacteria in the intestinal tract and improve the killing efficiency of nematodes. Our current findings may lay a theoretical foundation and open up new ideas for the development of ideal biocontrol agents.

## Supplementary Information

Below is the link to the electronic supplementary material.

Supplementary file1 (DOCX 4622 KB)

## Data Availability

The DDBJ/EMBL/GenBank accession number for the 16S rRNA gene sequence of strain CPCC 101271 is MT126327; The draft genome sequence of strain CPCC 101271 is WIAY00000000. The strain CPCC 101271 has been deposited in China Pharmaceutical Culture Collection and is available to the scientific research community without any special restrictions.
