# Supplementary material for: Stenotrophomonas strain CPCC 101271, an intestinal lifespan-prolonging bacterium for Caenorhabditis elegans that assists in host resistance to “Bacillus nematocida” colonization
Source: Arch Microbiol. 2021 Jul 14;203(8):4951–60. doi: 10.1007/s00203-021-02467-4 (PMC8502144; doi:10.1007/s00203-021-02467-4)

***Stenotrophomonas*** **strain CPCC 101271, an intestinal lifespan-prolonging** **bacterium for *Caenorhabditis elegans* that** **assists in host resistance to *“Bacillus nematocida”* colonization**

Rui Han^1#^, Yu Wang^1#^, Yang Deng^2^, Yuqin Zhang^2^, Lin Zhang^1*^, Qiuhong Niu^1*^

^1^College of Life Science and Agricultural Engineering, Nanyang Normal University, 1638 Wolong Road, Nanyang, Henan 473061, China

^2^Institute of Medicinal Biotechnology, Chinese Academy of Medical Sciences & Peking Union Medical College, Beijing, 100050, China

^#^These two authors Rui Han and Yu Wang contributed equally to the work.

*Corresponding author A: Lin Zhang

E-mail: zhanglin201207@163.com

*Corresponding author B: Qiuhong Niu

E-mail: [qiuhongniu723@163.com](mailto:qiuhongniu723@163.com)

Figure S1 Variation in the abundance of *Stenotrophomonas* and alpha-trehalose-phosphate synthase-encoding gene sequences based on metagenomic sequence analysis.


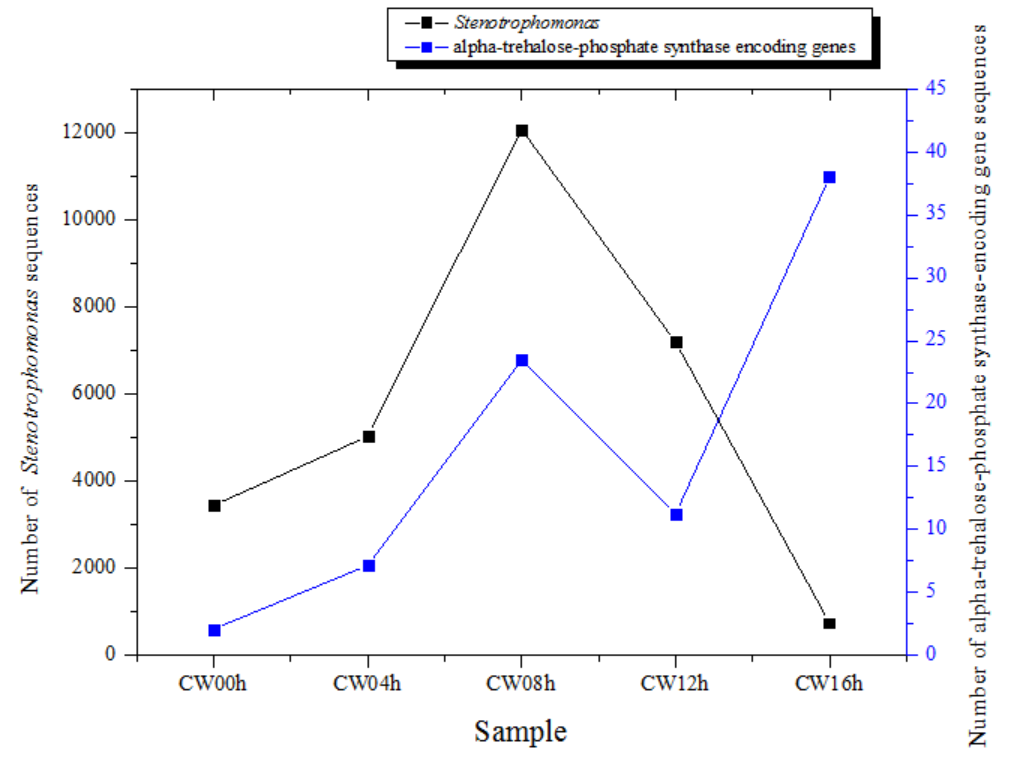


Figure S2 Variation in the abundance of *Bacillus* and trehalose-6-phosphate hydrolase-encoding gene sequences based on metagenomic sequence analysis


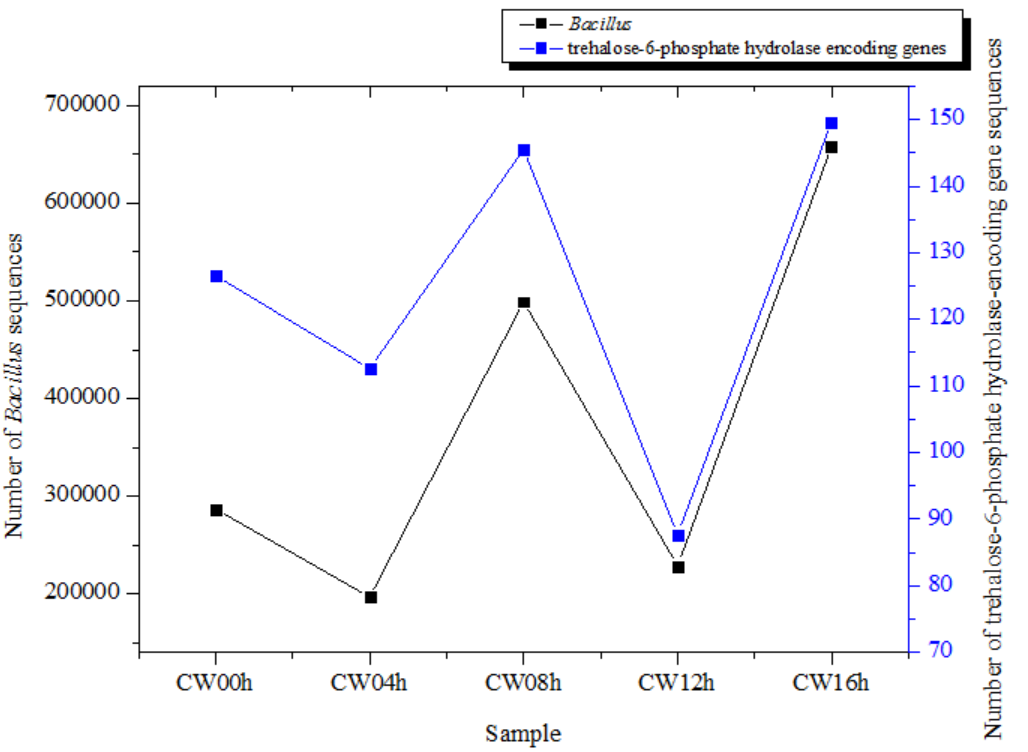

Supplement: Supplementary file 1 — Supplementary file1 (DOCX 4622 KB) [file 203_2021_2467_MOESM1_ESM.docx]
